# Supplementary material for: Gating the pore of the calcium-activated chloride channel TMEM16A
Source: Nat Commun. 2021 Feb 4;12:785. doi: 10.1038/s41467-020-20787-9 (PMC7862301; doi:10.1038/s41467-020-20787-9)
Supplement: Supplementary file 1 — Supplementary Information [file 41467_2020_20787_MOESM1_ESM.pdf]

# Supplementary Information

# Gating the pore of the calcium-activated chloride channel TMEM16A

Andy K. M. Lam, Jan Rheinberger, Cristina Paulino and Raimund Dutzler

## Supplementary Figures

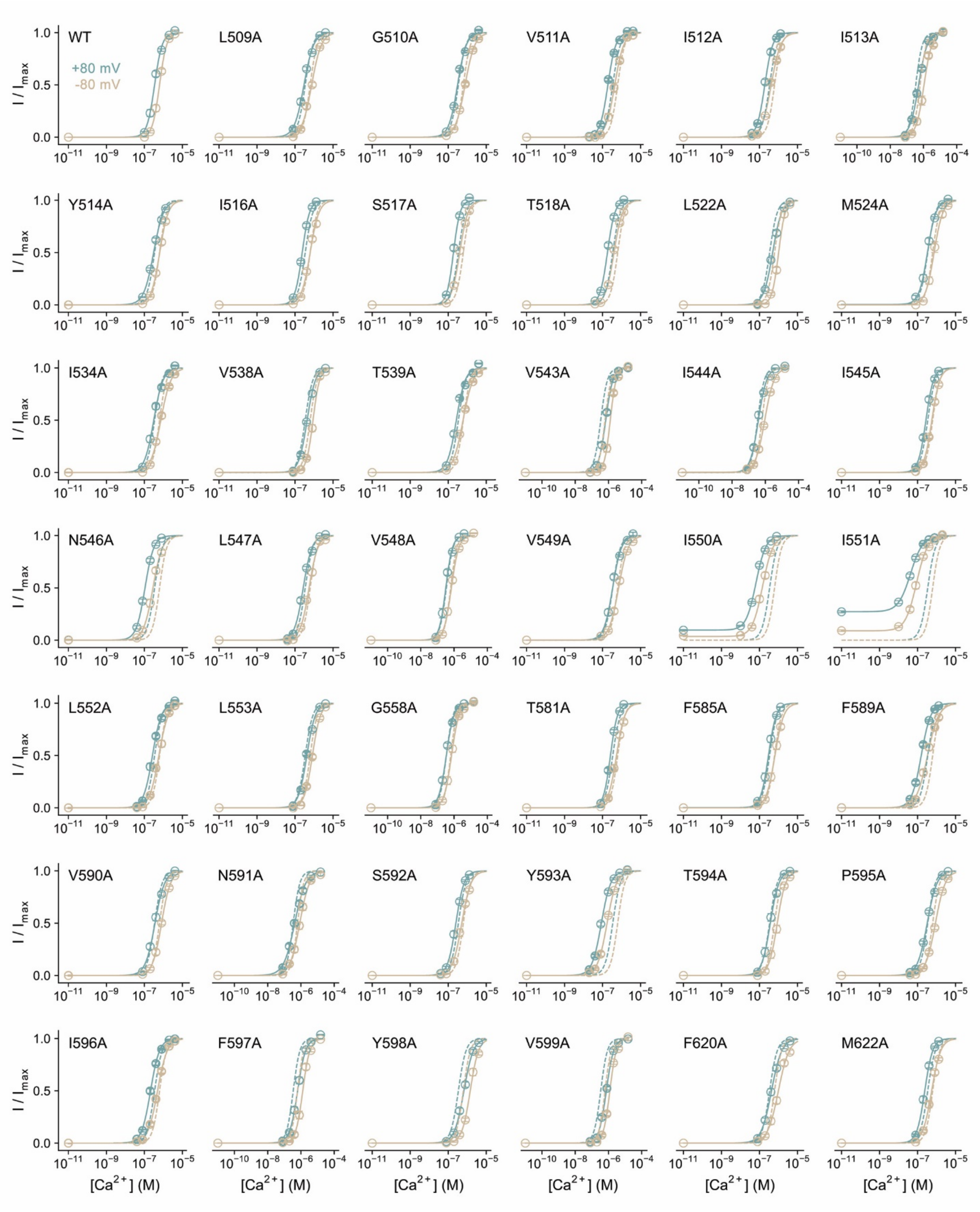

**Supplementary Fig. 1: Concentration-response relations of mutants I.** Relations were recorded at 80 and –80 mV. Data are averages of the indicated number of patches shown in Supplementary Tables 1 and 2, errors are SEM. Solid lines are fits to the Hill equation. Dashed lines are the relations of WT.

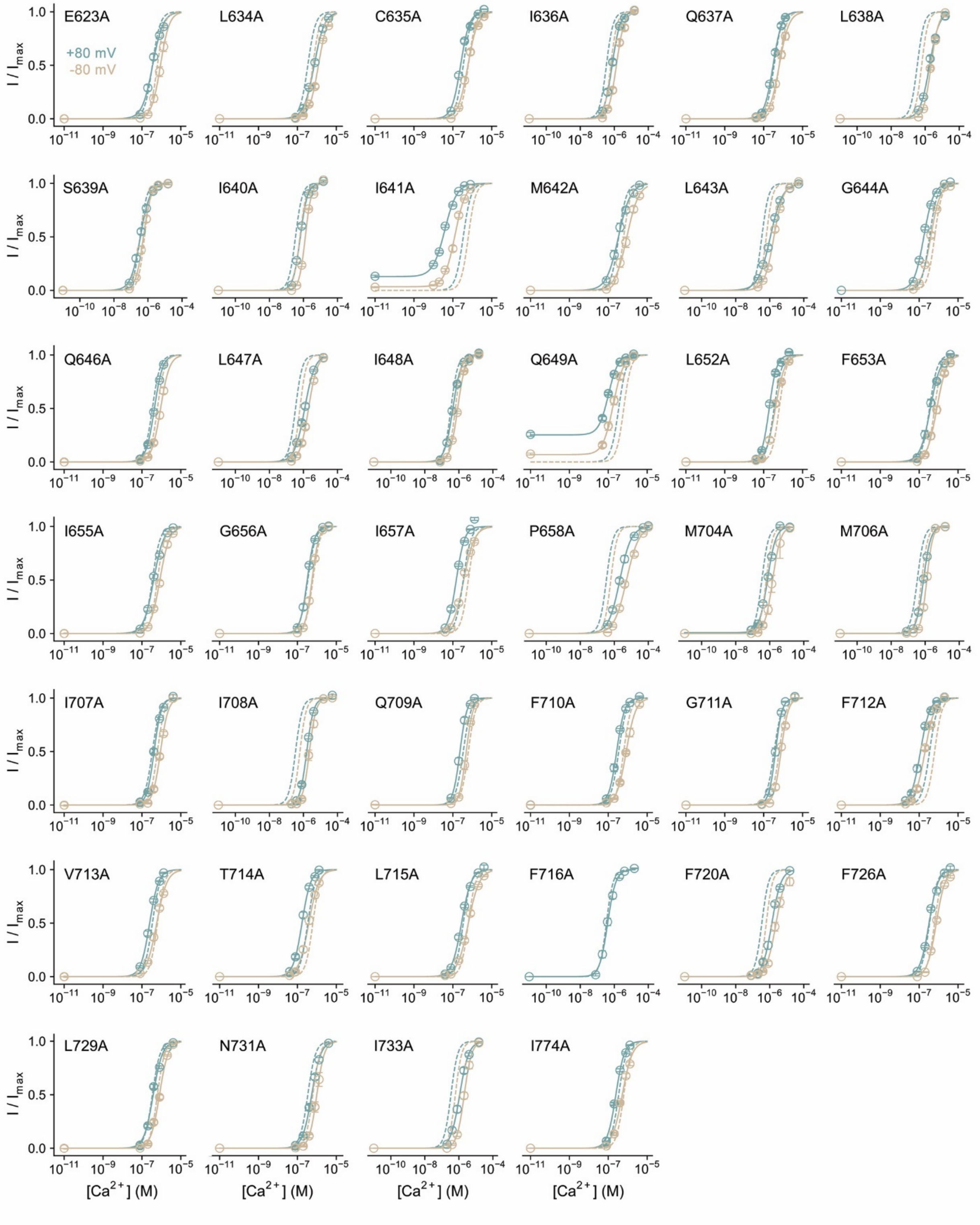
E623A
L634A
C635A
I636A
Q637A
L638A
S639A
I640A
I641A
M642A
L643A
G644A
Q646A
L647A
I648A
Q649A
L652A
F653A
I655A
G656A
I657A
P658A
M704A
M706A
I707A
I708A
Q709A
F710A
G711A
F712A
V713A
T714A
L715A
F716A
F720A
F726A
L729A
N731A
I733A
I774A
+80 mV
-80 mV
I / I_max
[Ca2+] (M)

**Supplementary Fig. 2: Concentration-response relations of mutants II.** Relations were recorded at 80 and –80 mV. Data are averages of the indicated number of patches shown in Supplementary Tables 2 and 3, errors are SEM. Solid lines are fits to the Hill equation. Dashed lines are the relations of WT.

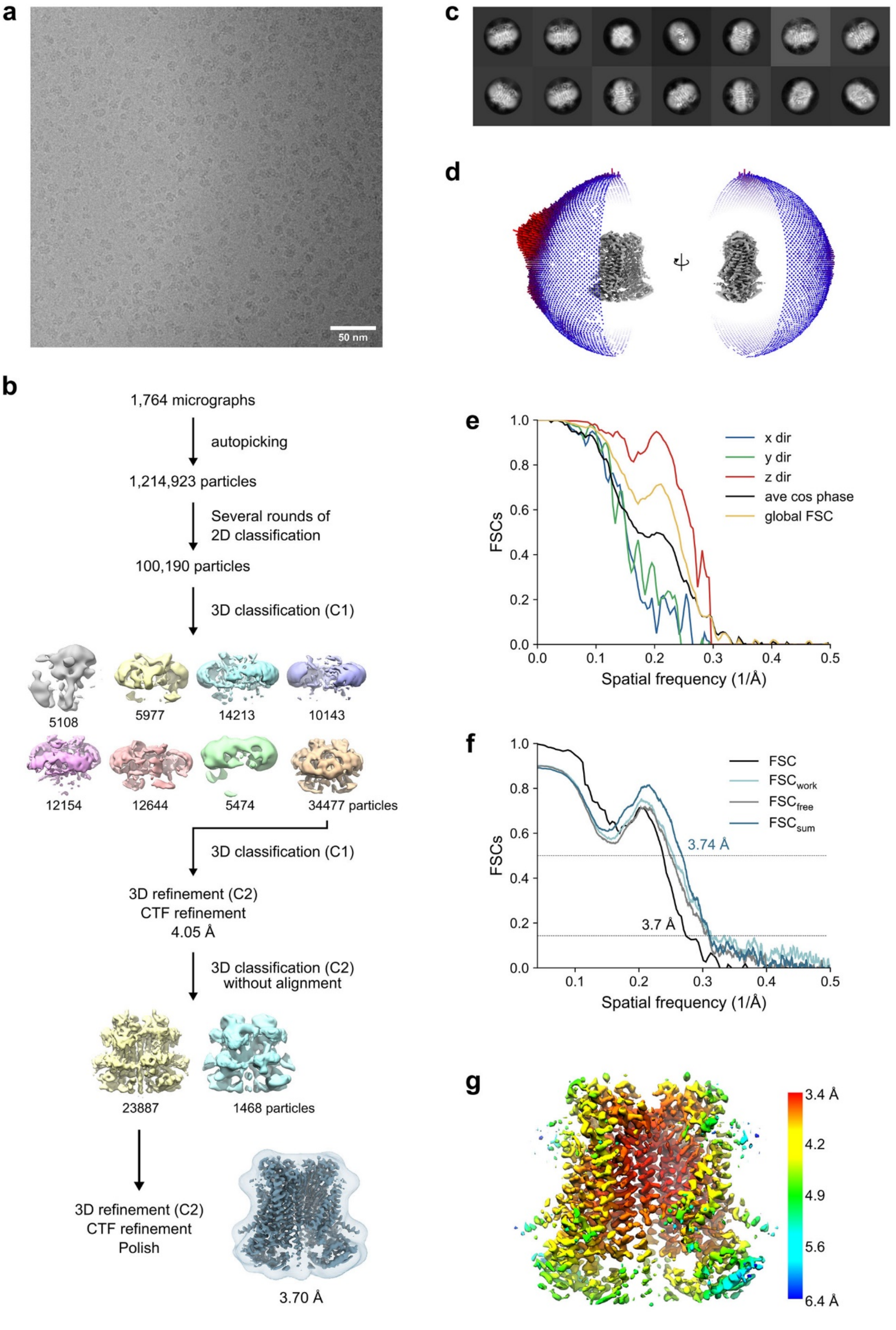

a
50 nm
b
1,764 micrographs
autopicking
1,214,923 particles
Several rounds of
2D classification
100,190 particles
3D classification (C1)
5108
5977
14213
10143
12154
12644
5474
34477 particles
3D classification (C1)
3D refinement (C2)
CTF refinement
4.05 Å
3D classification (C2)
without alignment
23887
1468 particles
3D refinement (C2)
CTF refinement
Polish
3.70 Å
c
d
e
FSCs
Spatial frequency (1/Å)
x dir
y dir
z dir
ave cos phase
global FSC
f
FSCs
Spatial frequency (1/Å)
FSC
FSC_work
FSC_free
FSC_sum
3.74 Å
3.7 Å
g
3.4 Å
4.2
4.9
5.6
6.4 Å

**Supplementary Fig. 3: Cryo-EM reconstruction of $Ca^{2+}$-bound wild-type mTMEM16A in the presence of diC8-PI(4,5)$P_2$.** **a**, Representative cryo-EM micrograph (scale bar: 50 nm). **b**, Workflow of single-particle processing. **c**, Selected 2D class averages. **d**, Representation of the angular distribution. **e**, Estimation of the directional FSCs, with the x-, y-, z- directions in blue, green, red, respectively. The global FSC is represented in yellow. The sphericity coefficient is 0.774. **f**, Model-vs-map and postprocess FSCs. **g**, Local resolution estimation of the final reconstruction colored based on the provided scale.

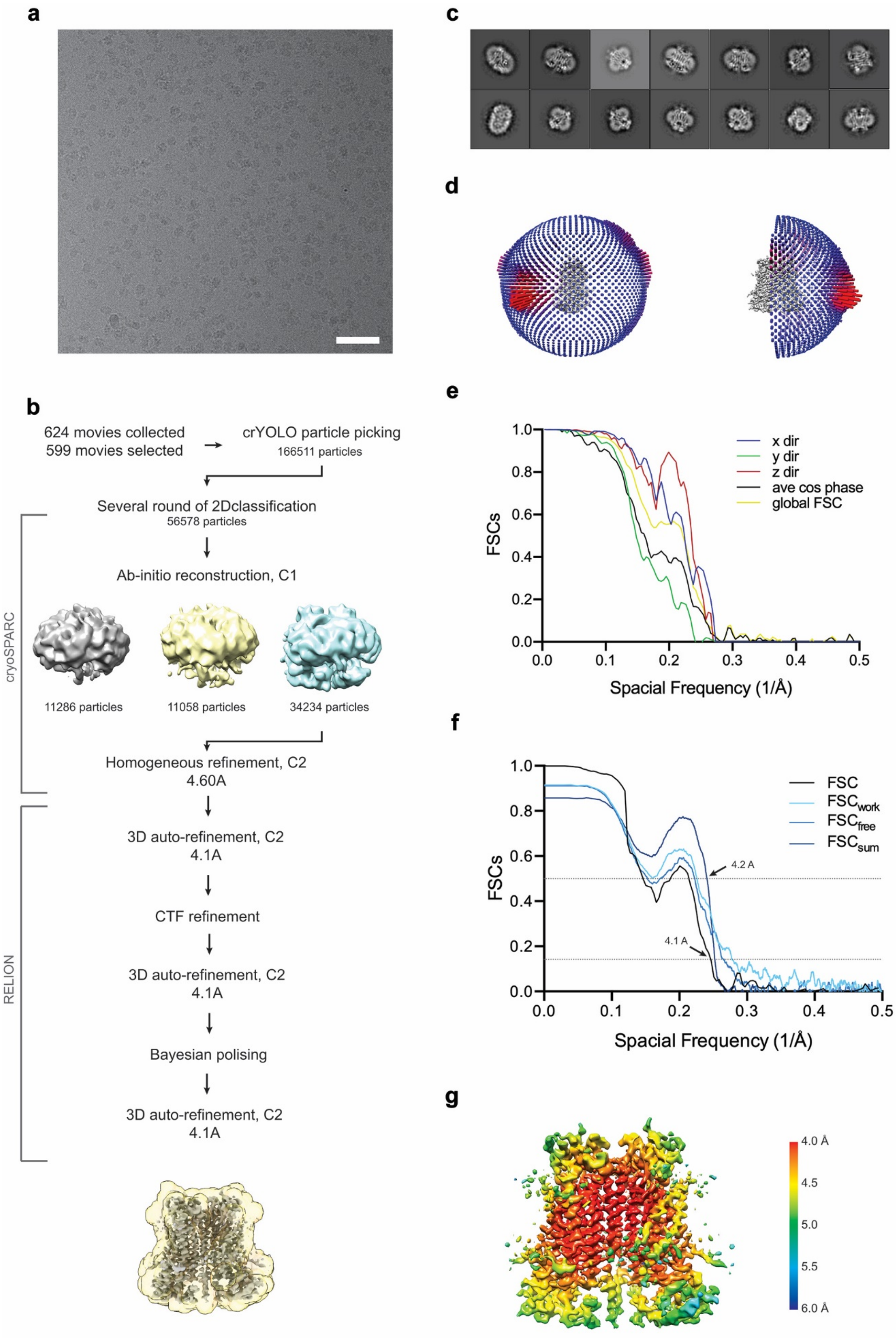
a
b
624 movies collected
599 movies selected
crYOLO particle picking
166511 particles
Several round of 2Dclassification
56578 particles
Ab-initio reconstruction, C1
cryoSPARC
11286 particles
11058 particles
34234 particles
Homogeneous refinement, C2
4.60A
RELION
3D auto-refinement, C2
4.1A
CTF refinement
3D auto-refinement, C2
4.1A
Bayesian polising
3D auto-refinement, C2
4.1A
c
d
e
x dir
y dir
z dir
ave cos phase
global FSC
FSCs
Spacial Frequency (1/Å)
f
FSC
FSCwork
FSCfree
FSCsum
4.2 A
4.1 A
FSCs
Spacial Frequency (1/Å)
g
4.0 Å
4.5
5.0
5.5
6.0 Å

**Supplementary Fig. 4: Cryo-EM reconstruction of $Ca^{2+}$-bound mTMEM16A-I551A in the presence of diC8-PI(4,5)$P_2$.** **a**, Representative cryo-EM micrograph (scale bar: 50 nm). **b**, Workflow of single-particle processing. **c**, Selected 2D class averages. **d**, Representation of the angular distribution. **e**, Estimation of the directional FSCs, with the x-, y-, z- directions in blue, green, red, respectively. The global FSC is represented in yellow. The sphericity coefficient is 0.980. **f**, Model-vs-map and postprocess FSCs. **g**, Local resolution estimation of the final reconstruction colored based on the provided scale.

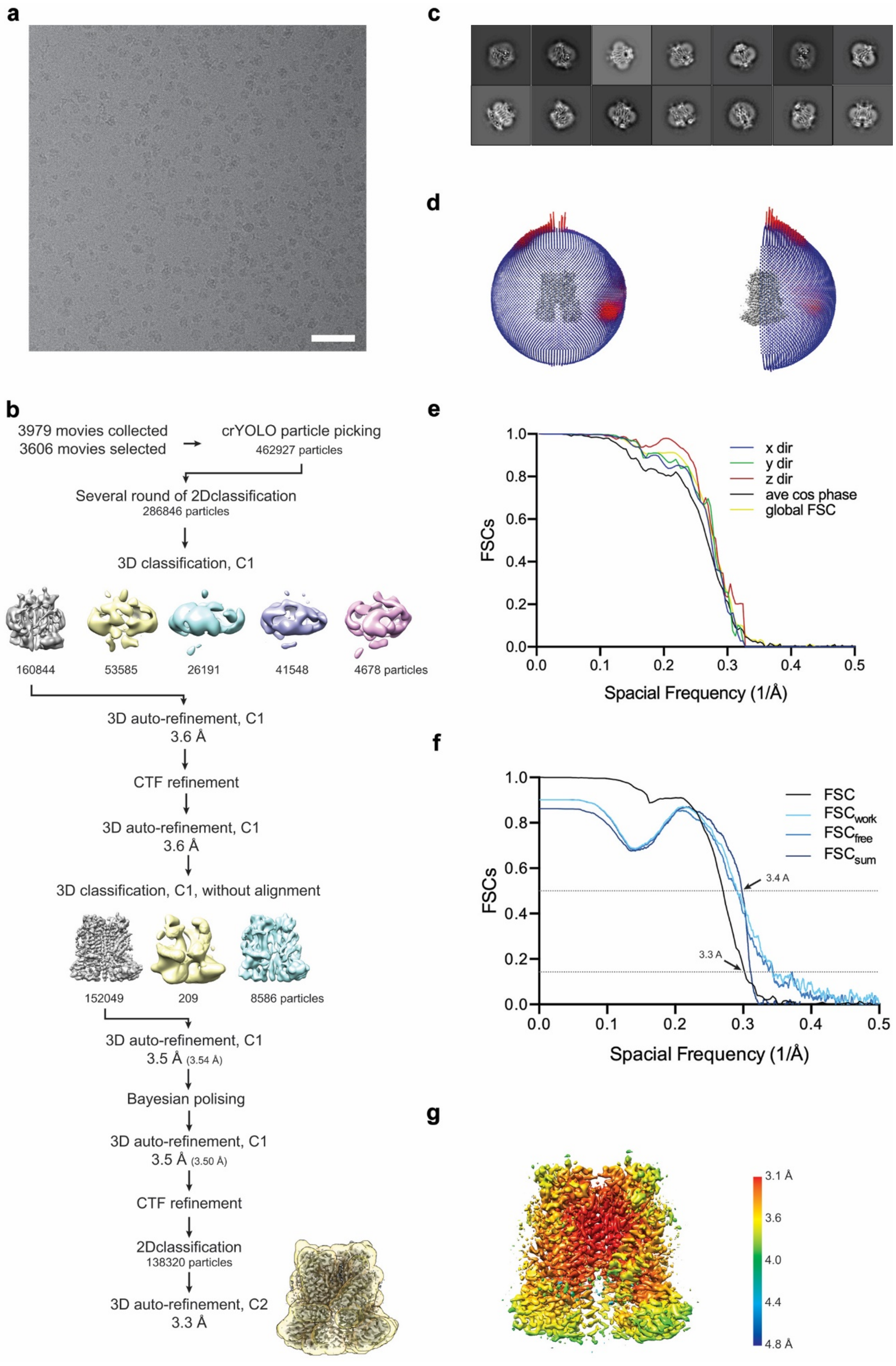

a
b
3979 movies collected
3606 movies selected
crYOLO particle picking
462927 particles
Several round of 2Dclassification
286846 particles
3D classification, C1
160844
53585
26191
41548
4678 particles
3D auto-refinement, C1
3.6 Å
CTF refinement
3D auto-refinement, C1
3.6 Å
3D classification, C1, without alignment
152049
209
8586 particles
3D auto-refinement, C1
3.5 Å (3.54 Å)
Bayesian polising
3D auto-refinement, C1
3.5 Å (3.50 Å)
CTF refinement
2Dclassification
138320 particles
3D auto-refinement, C2
3.3 Å
c
d
e
x dir
y dir
z dir
ave cos phase
global FSC
FSCs
Spacial Frequency (1/Å)
f
FSC
FSCwork
FSCfree
FSCsum
3.4 A
3.3 A
FSCs
Spacial Frequency (1/Å)
g
3.1 Å
3.6
4.0
4.4
4.8 Å

**Supplementary Fig. 5: Cryo-EM reconstruction of apo mTMEM16A-I551A in the presence of diC8-PI(4,5)$P_2$.** **a**, Representative cryo-EM micrograph (scale bar: 50 nm). **b**, Workflow of single-particle processing. **c**, Selected 2D class averages. **d**, Representation of the angular distribution. **e**, Estimation of the directional FSCs, with the x-, y-, z- directions in blue, green, red, respectively. The global FSC is represented in yellow. The sphericity coefficient is 0.770. **f**, Model-vs-map and postprocess FSCs. **g**, Local resolution estimation of the final reconstruction colored based on the provided scale.

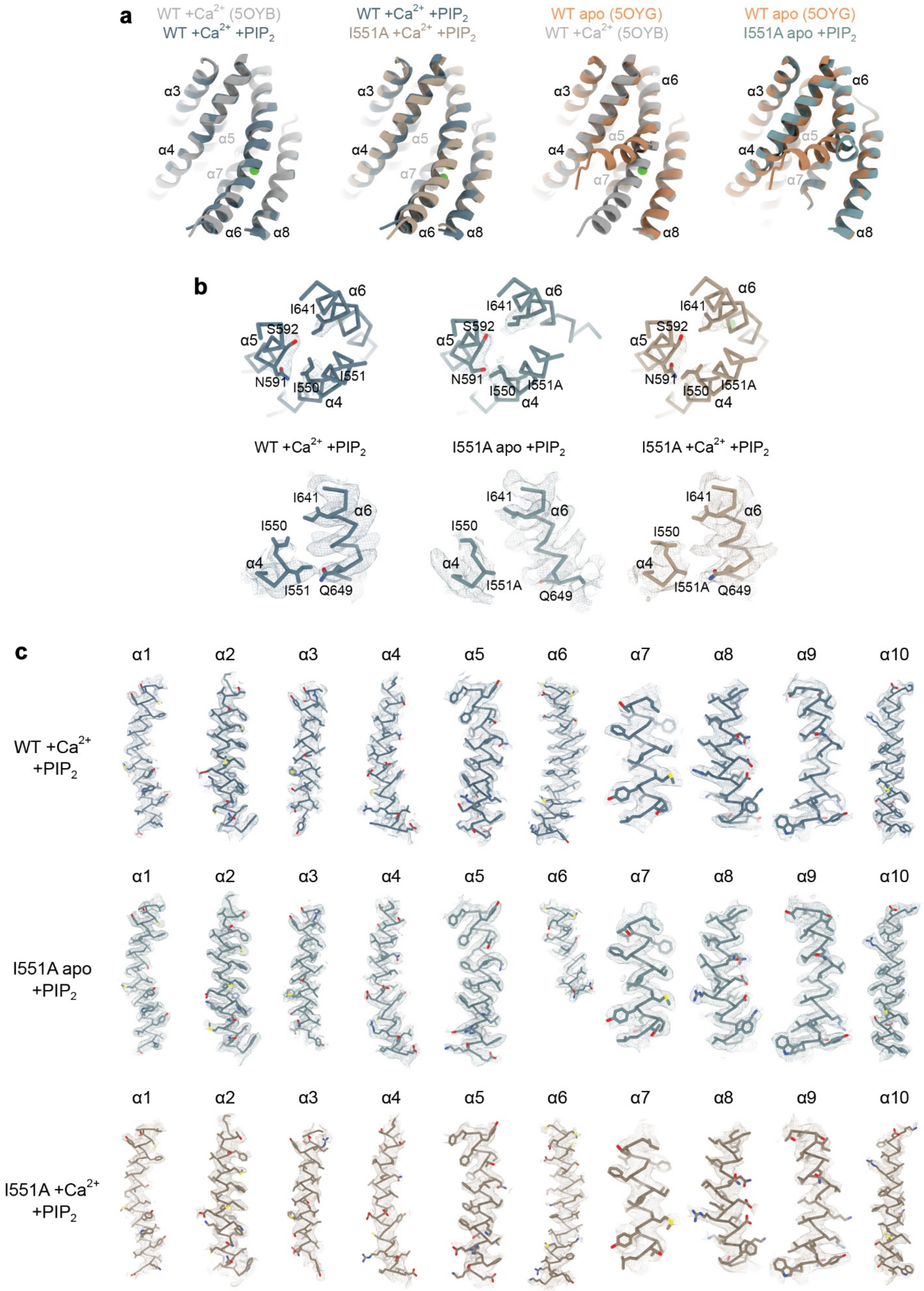

a
WT +Ca2+ (5OYB)
WT +Ca2+ +PIP2
WT +Ca2+ +PIP2
I551A +Ca2+ +PIP2
WT apo (5OYG)
WT +Ca2+ (5OYB)
WT apo (5OYG)
I551A apo +PIP2
α3
α4
α5
α6
α7
α8
b
I641
S592
N591
I550
I551
I551A
Q649
WT +Ca2+ +PIP2
I551A apo +PIP2
I551A +Ca2+ +PIP2
c
α1
α2
α3
α4
α5
α6
α7
α8
α9
α10
WT +Ca2+ +PIP2
I551A apo +PIP2
I551A +Ca2+ +PIP2

**Supplementary Fig. 6: Structural comparison and cryo-EM densities. a**, Superposition of the pore region of the indicated structures in ribbon representation. **b**, Gate region (top) and site of mutation (bottom) of the indicated structures in Cα representation with cryo-EM densities superimposed. (**a** and **b**) $Ca^{2+}$ ions in the $Ca^{2+}$-bound structures are shown as green spheres. **c**, Sections of cryo-EM densities of the transmembrane region superimposed on the refined models.

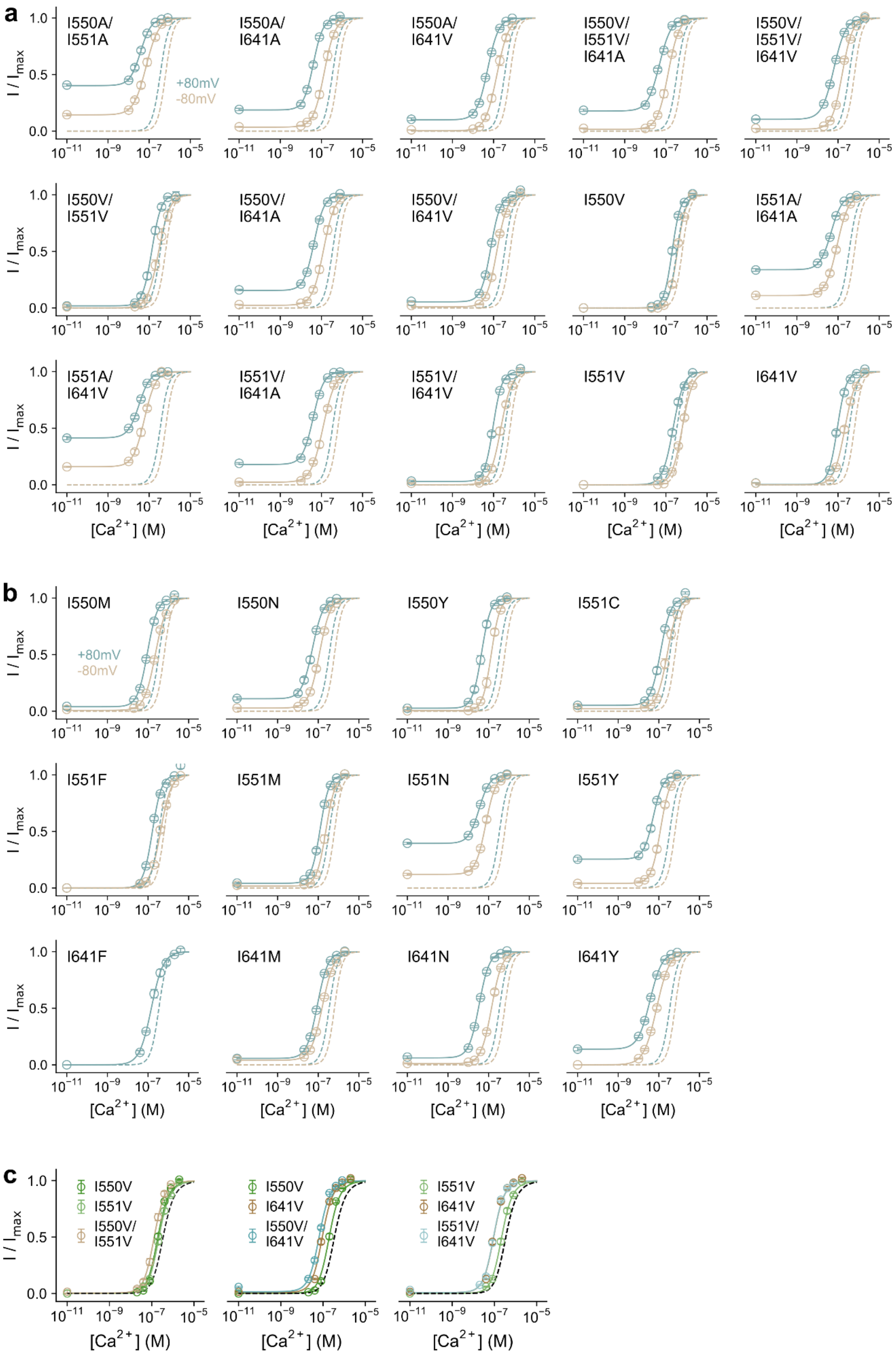
a
I550A/ I551A
I550A/ I641A
I550A/ I641V
I550V/ I551V/ I641A
I550V/ I551V/ I641V
I550V/ I551V
I550V/ I641A
I550V/ I641V
I550V
I551A/ I641A
I551A/ I641V
I551V/ I641A
I551V/ I641V
I551V
I641V
+80mV
-80mV
I / I_max
[Ca2+] (M)
b
I550M
I550N
I550Y
I551C
I551F
I551M
I551N
I551Y
I641F
I641M
I641N
I641Y
c
I550V
I551V
I550V/ I551V
I641V
I550V/ I641V
I551V/ I641V

**Supplementary Fig. 7: Concentration-response relations of mutants III.** **a**, Concentration-response relations of mutants displayed in Fig. 4b. **b**, Concentration-response relations of mutants displayed in Fig. 4d. (**a** and **b**) Relations were recorded at 80 and –80 mV. Data are averages of the indicated number of patches shown in Supplementary Tables 4 and 5 respectively, errors are SEM. Solid lines are fits to the Hill equation. Dashed lines are the relation of WT. **c**, Concentration-response relations of mutants selected for triple-mutant cycle analysis displayed in Fig. 5b. Data are averages of the indicated number of patches shown in Supplementary table 4, errors are SEM. Solid lines are from a global fit to an MWC-type gating model with a mutant-specific parameter ($\Delta G_{\mathrm{mut}}$) and shared binding constants (Eq. 4–6, and 9). Dashed lines are the relation of WT.

## Supplementary Tables

### Supplementary Table 1: Concentration-response parameters of alanine mutants I.

| | +80 mV | | | | –80 mV | | | | |
|---|---|---|---|---|---|---|---|---|---|
| | EC50 (μM) | CI | h | CI | EC50 (μM) | CI | h | CI | n |
| WT | 0.343 | 0.009 | 2.174 | 0.11 | 0.624 | 0.009 | 2.326 | 0.074 | 8 |
| L509A | 0.295 | 0.011 | 1.717 | 0.1 | 0.745 | 0.042 | 1.696 | 0.139 | 7 |
| G510A | 0.311 | 0.015 | 1.888 | 0.149 | 0.773 | 0.017 | 1.687 | 0.052 | 6 |
| V511A | 0.191 | 0.005 | 2.079 | 0.103 | 0.438 | 0.009 | 1.837 | 0.067 | 7 |
| I512A | 0.192 | 0.002 | 2.093 | 0.04 | 0.415 | 0.032 | 1.721 | 0.17 | 7 |
| I513A | 0.513 | 0.014 | 1.667 | 0.067 | 1.083 | 0.036 | 1.835 | 0.088 | 8 |
| Y514A | 0.305 | 0.012 | 1.67 | 0.094 | 0.638 | 0.027 | 1.917 | 0.088 | 7 |
| I516A | 0.238 | 0.003 | 2.259 | 0.055 | 0.623 | 0.059 | 1.881 | 0.189 | 7 |
| S517A | 0.189 | 0.005 | 2.491 | 0.15 | 0.404 | 0.019 | 1.981 | 0.145 | 6 |
| T518A | 0.18 | 0.003 | 2.214 | 0.078 | 0.399 | 0.017 | 1.934 | 0.129 | 7 |
| L522A | 0.496 | 0.013 | 1.826 | 0.079 | 1.024 | 0.02 | 2.64 | 0.106 | 9 |
| M524A | 0.333 | 0.009 | 1.939 | 0.096 | 0.738 | 0.025 | 2.007 | 0.123 | 6 |
| I534A | 0.31 | 0.016 | 1.778 | 0.146 | 0.73 | 0.043 | 1.637 | 0.134 | 5 |
| V538A | 0.431 | 0.009 | 1.987 | 0.073 | 0.882 | 0.019 | 2.648 | 0.141 | 9 |
| T539A | 0.269 | 0.016 | 1.87 | 0.189 | 0.605 | 0.026 | 1.646 | 0.107 | 6 |
| V543A | 0.684 | 0.006 | 2.017 | 0.034 | 1.284 | 0.023 | 2.468 | 0.077 | 7 |
| I544A | 0.381 | 0.017 | 1.747 | 0.121 | 0.92 | 0.017 | 1.542 | 0.037 | 5 |
| I545A | 0.276 | 0.005 | 2.183 | 0.076 | 0.565 | 0.021 | 2.091 | 0.106 | 7 |
| N546A | 0.107 | 0.002 | 1.885 | 0.043 | 0.282 | 0.014 | 1.754 | 0.112 | 6 |
| L547A | 0.269 | 0.01 | 1.909 | 0.119 | 0.545 | 0.02 | 1.89 | 0.119 | 6 |
| V548A | 0.319 | 0.026 | 2.183 | 0.342 | 0.688 | 0.019 | 1.951 | 0.095 | 6 |
| V549A | 0.339 | 0.012 | 1.887 | 0.118 | 0.697 | 0.014 | 1.718 | 0.051 | 7 |
| I550A | 0.066 | 0.002 | 1.667 | 0.058 | 0.147 | 0.005 | 1.6 | 0.07 | 7 |
| I551A | 0.043 | 0.001 | 1.347 | 0.031 | 0.088 | 0.003 | 1.48 | 0.062 | 5 |
| L552A | 0.265 | 0.009 | 1.853 | 0.109 | 0.576 | 0.022 | 1.758 | 0.11 | 7 |
| L553A | 0.418 | 0.019 | 1.957 | 0.155 | 0.817 | 0.025 | 2.046 | 0.111 | 5 |
| G558A | 0.341 | 0.008 | 1.907 | 0.079 | 0.702 | 0.011 | 1.92 | 0.053 | 8 |
| T581A | 0.26 | 0.004 | 2.358 | 0.08 | 0.54 | 0.043 | 1.979 | 0.201 | 7 |
| F585A | 0.299 | 0.002 | 2.135 | 0.033 | 0.654 | 0.027 | 1.864 | 0.078 | 10 |
| F589A | 0.156 | 0.001 | 1.729 | 0.012 | 0.335 | 0.024 | 1.467 | 0.117 | 6 |
| V590A | 0.361 | 0.012 | 1.716 | 0.09 | 0.799 | 0.009 | 1.838 | 0.031 | 7 |
| N591A | 0.422 | 0.012 | 1.314 | 0.042 | 0.848 | 0.017 | 1.475 | 0.042 | 7 |

h, Hill coefficient; n, number of patches; CI, 95% confidence interval

**Supplementary Table 2: Concentration-response parameters of alanine mutants II.**

| | +80 mV | | | | –80 mV | | | | |
|---|---|---|---|---|---|---|---|---|---|
| | EC50 (µM) | CI | h | CI | EC50 (µM) | CI | h | CI | n |
| S592A | 0.254 | 0.004 | 2.011 | 0.06 | 0.519 | 0.035 | 1.783 | 0.134 | 7 |
| Y593A | 0.087 | 0.003 | 1.765 | 0.073 | 0.172 | 0.004 | 1.745 | 0.057 | 7 |
| T594A | 0.374 | 0.01 | 1.917 | 0.089 | 0.866 | 0.027 | 1.974 | 0.103 | 7 |
| P595A | 0.322 | 0.009 | 1.69 | 0.078 | 0.872 | 0.013 | 1.675 | 0.033 | 7 |
| I596A | 0.21 | 0.005 | 1.843 | 0.071 | 0.49 | 0.007 | 1.709 | 0.036 | 8 |
| F597A | 0.63 | 0.015 | 1.816 | 0.072 | 1.285 | 0.023 | 2.052 | 0.056 | 8 |
| Y598A | 0.672 | 0.018 | 1.748 | 0.074 | 1.441 | 0.067 | 1.984 | 0.119 | 7 |
| V599A | 0.744 | 0.013 | 1.963 | 0.061 | 1.213 | 0.026 | 2.226 | 0.078 | 8 |
| F620A | 0.432 | 0.01 | 1.612 | 0.053 | 0.993 | 0.046 | 1.53 | 0.078 | 9 |
| M622A | 0.24 | 0.003 | 2.091 | 0.056 | 0.553 | 0.038 | 1.828 | 0.142 | 8 |
| E623A | 0.354 | 0.042 | 1.619 | 0.24 | 0.895 | 0.09 | 1.904 | 0.15 | 4 |
| L634A | 0.729 | 0.021 | 1.629 | 0.067 | 1.206 | 0.071 | 1.809 | 0.131 | 7 |
| C635A | 0.25 | 0.01 | 1.931 | 0.126 | 0.596 | 0.024 | 1.725 | 0.108 | 7 |
| I636A | 0.745 | 0.012 | 1.833 | 0.048 | 1.329 | 0.051 | 1.913 | 0.108 | 8 |
| Q637A | 0.304 | 0.006 | 1.94 | 0.07 | 0.632 | 0.035 | 1.735 | 0.089 | 7 |
| L638A | 1.818 | 0.062 | 1.564 | 0.069 | 2.243 | 0.074 | 1.979 | 0.122 | 8 |
| S639A | 0.358 | 0.008 | 1.536 | 0.044 | 0.538 | 0.009 | 1.934 | 0.057 | 7 |
| I640A | 0.658 | 0.017 | 2.07 | 0.1 | 1.29 | 0.045 | 2.425 | 0.147 | 7 |
| I641A | 0.039 | 0.001 | 1.405 | 0.05 | 0.129 | 0.005 | 1.582 | 0.077 | 10 |
| M642A | 0.335 | 0.019 | 1.408 | 0.099 | 0.906 | 0.05 | 1.418 | 0.081 | 5 |
| L643A | 1.085 | 0.017 | 1.218 | 0.019 | 1.763 | 0.045 | 1.555 | 0.054 | 8 |
| G644A | 0.174 | 0.005 | 1.734 | 0.071 | 0.395 | 3.351 | 1.775 | 27.167 | 6 |
| Q646A | 0.436 | 0.009 | 2.044 | 0.069 | 0.91 | 0.033 | 1.901 | 0.053 | 7 |
| L647A | 1.194 | 0.026 | 1.459 | 0.042 | 2.003 | 0.023 | 1.699 | 0.024 | 8 |
| I648A | 0.445 | 0.013 | 1.795 | 0.082 | 0.849 | 0.015 | 1.95 | 0.058 | 7 |
| Q649A | 0.101 | 0.003 | 1.746 | 0.079 | 0.18 | 0.003 | 1.638 | 0.037 | 6 |
| L652A | 0.191 | 0.006 | 2.247 | 0.133 | 0.397 | 0.021 | 1.8 | 0.148 | 7 |
| F653A | 0.378 | 0.014 | 1.664 | 0.096 | 0.813 | 0.021 | 1.714 | 0.062 | 9 |
| I655A | 0.4 | 0.017 | 1.693 | 0.113 | 0.833 | 0.025 | 1.772 | 0.078 | 6 |
| G656A | 0.321 | 0.004 | 2.335 | 0.055 | 0.563 | 0.005 | 2.532 | 0.053 | 8 |
| I657A | 0.151 | 0.009 | 2.037 | 0.2 | 0.362 | 0.028 | 1.566 | 0.143 | 7 |
| P658A | 2.114 | 0.061 | 1.309 | 0.045 | 5.241 | 0.434 | 1.202 | 0.105 | 6 |
| M704A | 0.74 | 0.026 | 1.788 | 0.106 | 1.486 | 0.042 | 1.855 | 0.083 | 7 |
| M706A | 0.796 | 0.011 | 2.051 | 0.057 | 1.227 | 0.01 | 2.767 | 0.068 | 7 |

h, Hill coefficient; n, number of patches; CI, 95% confidence interval

**Supplementary Table 3: Concentration-response parameters of alanine mutants III.**

| | +80 mV | | | | –80 mV | | | | |
|---|---|---|---|---|---|---|---|---|---|
| | EC50 (µM) | CI | h | CI | EC50 (µM) | CI | h | CI | n |
| I707A | 0.419 | 0.012 | 2.335 | 0.134 | 0.898 | 0.028 | 2.188 | 0.14 | 7 |
| I708A | 1.567 | 0.026 | 2.149 | 0.061 | 2.246 | 0.061 | 2.239 | 0.132 | 7 |
| Q709A | 0.232 | 0.004 | 2.44 | 0.089 | 0.493 | 0.012 | 2.653 | 0.13 | 4 |
| F710A | 0.27 | 0.006 | 2.138 | 0.083 | 0.838 | 0.017 | 1.781 | 0.058 | 4 |
| G711A | 0.404 | 0.003 | 2.782 | 0.051 | 0.776 | 0.02 | 2.341 | 0.123 | 9 |
| F712A | 0.117 | 0.003 | 1.813 | 0.059 | 0.2 | 0.008 | 1.733 | 0.099 | 7 |
| V713A | 0.249 | 0.004 | 1.918 | 0.057 | 0.574 | 0.037 | 1.644 | 0.099 | 7 |
| T714A | 0.172 | 0.003 | 1.937 | 0.057 | 0.387 | 0.015 | 1.675 | 0.082 | 7 |
| L715A | 0.28 | 0.009 | 1.766 | 0.088 | 0.628 | 0.01 | 1.529 | 0.033 | 7 |
| F716A | 0.409 | 0.012 | 1.827 | 0.087 | n/a | n/a | n/a | n/a | 6 |
| F720A | 1.338 | 0.046 | 1.604 | 0.071 | 2.366 | 0.173 | 1.534 | 0.152 | 6 |
| F726A | 0.322 | 0.014 | 1.871 | 0.139 | 0.76 | 0.028 | 1.896 | 0.117 | 7 |
| L729A | 0.38 | 0.02 | 1.833 | 0.162 | 0.798 | 0.035 | 1.95 | 0.144 | 7 |
| N731A | 0.544 | 0.011 | 1.847 | 0.058 | 0.994 | 0.031 | 2.234 | 0.061 | 8 |
| I733A | 1.12 | 0.025 | 1.627 | 0.047 | 2.075 | 0.023 | 1.971 | 0.04 | 8 |
| I774A | 0.246 | 0.009 | 2.001 | 0.127 | 0.524 | 0.075 | 1.668 | 0.243 | 6 |

h, Hill coefficient; n, number of patches; CI, 95% confidence interval; n/a not applicable

**Supplementary Table 4: Concentration-response parameters of mutants (methyl series).**

| | +80 mV | | | | –80 mV | | | | |
|---|---|---|---|---|---|---|---|---|---|
| | EC50 (μM) | CI | h | CI | EC50 (μM) | CI | h | CI | n |
| I550A/I551A | 0.038 | 0.001 | 1.666 | 0.045 | 0.071 | 0.002 | 1.417 | 0.04 | 6 |
| I550A/I641A | 0.04 | 0.001 | 1.856 | 0.073 | 0.137 | 0.002 | 1.65 | 0.032 | 6 |
| I550A/I641V | 0.051 | 0.001 | 1.631 | 0.041 | 0.145 | 0.002 | 1.549 | 0.023 | 7 |
| I550V/I551V/I641A | 0.049 | 0.002 | 1.511 | 0.064 | 0.128 | 0.003 | 1.649 | 0.049 | 8 |
| I550V/I551V/I641V | 0.062 | 0.001 | 1.493 | 0.019 | 0.16 | 0.006 | 1.752 | 0.089 | 6 |
| I550V/I551V | 0.135 | 0.001 | 1.932 | 0.023 | 0.289 | 0.01 | 1.618 | 0.081 | 8 |
| I550V/I641A | 0.044 | 0.001 | 1.746 | 0.056 | 0.126 | 0.002 | 1.619 | 0.037 | 6 |
| I550V/I641V | 0.072 | 0.003 | 1.86 | 0.105 | 0.144 | 0.005 | 1.73 | 0.081 | 6 |
| I550V | 0.201 | 0.004 | 2.165 | 0.076 | 0.365 | 0.013 | 2.113 | 0.143 | 6 |
| I551A/I641A | 0.046 | 0.001 | 1.609 | 0.033 | 0.09 | 0.001 | 1.562 | 0.011 | 6 |
| I551A/I641V | 0.032 | 0.001 | 1.614 | 0.03 | 0.058 | 0.002 | 1.591 | 0.086 | 7 |
| I551V/I641A | 0.039 | 0.001 | 1.682 | 0.061 | 0.121 | 0.005 | 1.481 | 0.071 | 6 |
| I551V/I641V | 0.094 | 0.003 | 2.05 | 0.105 | 0.225 | 0.008 | 1.665 | 0.089 | 6 |
| I551V | 0.225 | 0.006 | 1.694 | 0.064 | 0.622 | 0.025 | 1.704 | 0.086 | 9 |
| I641V | 0.092 | 0.003 | 2.012 | 0.109 | 0.215 | 0.008 | 1.652 | 0.083 | 8 |

h, Hill coefficient; n, number of patches; CI, 95% confidence interval

**Supplementary Table 5: Concentration-response parameters of mutants ($\Delta G_{hydration}$ series).**

| | +80 mV | | | | –80 mV | | | | |
|---|---|---|---|---|---|---|---|---|---|
| | EC50 (μM) | CI | h | CI | EC50 (μM) | CI | h | CI | n |
| I550M | 0.094 | 0.003 | 1.784 | 0.075 | 0.231 | 0.007 | 1.602 | 0.067 | 5 |
| I550N | 0.052 | 0.001 | 1.638 | 0.035 | 0.115 | 0.002 | 1.651 | 0.042 | 7 |
| I550Y | 0.045 | 0.001 | 1.957 | 0.037 | 0.135 | 0.002 | 2.07 | 0.044 | 7 |
| I551C | 0.121 | 0.005 | 1.756 | 0.097 | 0.261 | 0.007 | 1.698 | 0.072 | 7 |
| I551F | 0.164 | 0.01 | 1.931 | 0.185 | 0.415 | 0.013 | 1.693 | 0.085 | 7 |
| I551M | 0.123 | 0.002 | 1.989 | 0.041 | 0.237 | 0.006 | 1.908 | 0.081 | 7 |
| I551N | 0.034 | 0.001 | 1.665 | 0.035 | 0.072 | 0.001 | 1.746 | 0.033 | 7 |
| I551Y | 0.053 | 0.001 | 1.825 | 0.049 | 0.121 | 0.001 | 1.908 | 0.033 | 8 |
| I641F | 0.146 | 0.005 | 1.483 | 0.052 | n/a | n/a | n/a | n/a | 7 |
| I641M | 0.09 | 0.001 | 1.66 | 0.03 | 0.161 | 0.004 | 1.545 | 0.047 | 7 |
| I641N | 0.034 | 0.001 | 1.725 | 0.04 | 0.14 | 0.001 | 1.678 | 0.02 | 7 |
| I641Y | 0.037 | 0.001 | 1.458 | 0.011 | 0.077 | 0.001 | 1.375 | 0.02 | 7 |

h, Hill coefficient; n, number of patches; n/a, not applicable; CI, 95% confidence interval

**Supplementary Table 6: List of primers.**

| Name | F/R | Sequence (5'-to-3') |
|---|---|---|
| L509A | F | ATC GTC GCC GGA GTT ATC ATC TAT AGA ATC TCC ACA GCT |
| L509A | R | AAC TCC GGC GAC GAT TGC AAA TGT CAC TGC GAT CAT GAA |
| G510A | F | GTC CTC GCC GTT ATC ATC TAT AGA ATC TCC ACA GCT GCA |
| G510A | R | GAT AAC GGC GAG GAC GAT TGC AAA TGT CAC TGC GAT CAT |
| V511A | F | CTC GGA GCC ATC ATC TAT AGA ATC TCC ACA GCT GCA GCC |
| V511A | R | GAT GAT GGC TCC GAG GAC GAT TGC AAA TGT CAC TGC GAT |
| I512A | F | GGA GTT GCC ATC TAT AGA ATC TCC ACA GCT GCA GCC TTG |
| I512A | R | ATA GAT GGC AAC TCC GAG GAC GAT TGC AAA TGT CAC TGC |
| I513A | F | GTT ATC GCC TAT AGA ATC TCC ACA GCT GCA GCC TTG GCC |
| I513A | R | TCT ATA GGC GAT AAC TCC GAG GAC GAT TGC AAA TGT CAC |
| I516A | F | TAT AGA GCC TCC ACA GCT GCA GCC TTG GCC ATG AAC TCC |
| I516A | R | TGT GGA GGC TCT ATA GAT GAT AAC TCC GAG GAC GAT TGC |
| S517A | F | AGA ATC GCC ACA GCT GCA GCC TTG GCC ATG AAC TCC TCC |
| S517A | R | AGC TGT GGC GAT TCT ATA GAT GAT AAC TCC GAG GAC GAT |
| T518A | F | ATC TCC GCC GCT GCA GCC TTG GCC ATG AAC TCC TCC CCG |
| T518A | R | TGC AGC GGC GGA GAT TCT ATA GAT GAT AAC TCC GAG GAC |
| L522A | F | GCA GCC GCC GCC ATG AAC TCC TCC CCG TCT GTG CGG TCC |
| L522A | R | CAT GGC GGC GGC TGC AGC TGT GGA GAT TCT ATA GAT GAT |
| M524A | F | TTG GCC GCC AAC TCC TCC CCG TCT GTG CGG TCC AAC ATC |
| M524A | R | GGA GTT GGC GGC CAA GGC TGC AGC TGT GGA GAT TCT ATA |
| I534A | F | TCC AAC GCC CGG GTT ACA GTC ACG GCC ACC GCT GTT ATC |
| I534A | R | AAC CCG GGC GTT GGA CCG CAC AGA CGG GGA GGA GTT CAT |
| V538A | F | GTT ACA GCC ACG GCC ACC GCT GTT ATC ATC AAC CTC GTG |
| V538A | R | GGC CGT GGC TGT AAC CCG GAT GTT GGA CCG CAC AGA CGG |
| T539A | F | ACA GTC GCC GCC ACC GCT GTT ATC ATC AAC CTC GTG GTC |
| T539A | R | GGT GGC GGC GAC TGT AAC CCG GAT GTT GGA CCG CAC AGA |
| V543A | F | ACC GCT GCC ATC ATC AAC CTC GTG GTC ATC ATT CTG CTG |
| V543A | R | GAT GAT GGC AGC GGT GGC CGT GAC TGT AAC CCG GAT GTT |
| I544A | F | GCT GTT GCC ATC AAC CTC GTG GTC ATC ATT CTG CTG GAT |
| I544A | R | GTT GAT GGC AAC AGC GGT GGC CGT GAC TGT AAC CCG GAT |
| I545A | F | GTT ATC GCC AAC CTC GTG GTC ATC ATT CTG CTG GAT GAA |
| I545A | R | GAG GTT GGC GAT AAC AGC GGT GGC CGT GAC TGT AAC CCG |
| N546A | F | ATC ATC GCA CTC GTG GTC ATC ATT CTG CTG GAT GAA GTT |
| N546A | R | CAC GAG TGC GAT GAT AAC AGC GGT GGC CGT GAC TGT AAC |
| V548A | F | AAC CTC GCC GTC ATC ATT CTG CTG GAT GAA GTT TAC GGC |
| V548A | R | GAT GAC GGC GAG GTT GAT GAT AAC AGC GGT GGC CGT GAC |
| V549A | F | CTC GTG GCC ATC ATT CTG CTG GAT GAA GTT TAC GGC TGC |
| V549A | R | AAT GAT GGC CAC GAG GTT GAT GAT AAC AGC GGT GGC CGT |
| I550A | F | GTG GTC GCC ATT CTG CTG GAT GAA GTT TAC GGC TGC ATT |

| | | |
|---|---|---|
| I550A | R | CAG AAT GGC GAC CAC GAG GTT GAT GAT AAC AGC GGT GGC |
| I550A/I551A | F | GTC GCC GCC CTG CTG GAT GAA GTT TAC GGC TGC ATT GCC |
| I550A/I551A | R | CAG CAG GGC GGC GAC CAC GAG GTT GAT GAT AAC AGC GGT |
| I550C | F | GTG GTC TGT ATT CTG CTG GAT GAA GTT TAC GGC TGC ATT |
| I550C | R | CAG AAT ACA GAC CAC GAG GTT GAT GAT AAC AGC GGT GGC |
| I550F | F | GTG GTC TTT ATT CTG CTG GAT GAA GTT TAC GGC TGC ATT |
| I550F | R | CAG AAT AAA GAC CAC GAG GTT GAT GAT AAC AGC GGT GGC |
| I550M | F | GTG GTC ATG ATT CTG CTG GAT GAA GTT TAC GGC TGC ATT |
| I550M | R | CAG AAT CAT GAC CAC GAG GTT GAT GAT AAC AGC GGT GGC |
| I550N | F | GTG GTC AAC ATT CTG CTG GAT GAA GTT TAC GGC TGC ATT |
| I550N | R | CAG AAT GTT GAC CAC GAG GTT GAT GAT AAC AGC GGT GGC |
| I550V | F | GTG GTC GTG ATT CTG CTG GAT GAA GTT TAC GGC TGC ATT |
| I550V | R | CAG AAT CAC GAC CAC GAG GTT GAT GAT AAC AGC GGT GGC |
| I550V/I551V | F | GTC GTG GTG CTG CTG GAT GAA GTT TAC GGC TGC ATT GCC |
| I550V/I551V | R | CAG CAG CAC CAC GAC CAC GAG GTT GAT GAT AAC AGC GGT |
| I550Y | F | GTG GTC TAC ATT CTG CTG GAT GAA GTT TAC GGC TGC ATT |
| I550Y | R | CAG AAT GTA GAC CAC GAG GTT GAT GAT AAC AGC GGT GGC |
| I551A | F | GTC ATC GCC CTG CTG GAT GAA GTT TAC GGC TGC ATT GCC |
| I551A | R | CAG CAG GGC GAT GAC CAC GAG GTT GAT GAT AAC AGC GGT |
| I551C | F | GTC ATC TGT CTG CTG GAT GAA GTT TAC GGC TGC ATT GCC |
| I551C | R | CAG CAG ACA GAT GAC CAC GAG GTT GAT GAT AAC AGC GGT |
| I551F | F | GTC ATC TTT CTG CTG GAT GAA GTT TAC GGC TGC ATT GCC |
| I551F | R | CAG CAG AAA GAT GAC CAC GAG GTT GAT GAT AAC AGC GGT |
| I551M | F | GTC ATC ATG CTG CTG GAT GAA GTT TAC GGC TGC ATT GCC |
| I551M | R | CAG CAG CAT GAT GAC CAC GAG GTT GAT GAT AAC AGC GGT |
| I551N | F | GTC ATC AAC CTG CTG GAT GAA GTT TAC GGC TGC ATT GCC |
| I551N | R | CAG CAG GTT GAT GAC CAC GAG GTT GAT GAT AAC AGC GGT |
| I551V | F | GTC ATC GTG CTG CTG GAT GAA GTT TAC GGC TGC ATT GCC |
| I551V | R | CAG CAG CAC GAT GAC CAC GAG GTT GAT GAT AAC AGC GGT |
| I551Y | F | GTC ATC TAC CTG CTG GAT GAA GTT TAC GGC TGC ATT GCC |
| I551Y | R | CAG CAG GTA GAT GAC CAC GAG GTT GAT GAT AAC AGC GGT |
| L552A | F | ATC ATT GCC CTG GAT GAA GTT TAC GGC TGC ATT GCC AGG |
| L552A | R | ATC CAG GGC AAT GAT GAC CAC GAG GTT GAT GAT AAC AGC |
| L553A | F | ATT CTG GCC GAT GAA GTT TAC GGC TGC ATT GCC AGG TGG |
| L553A | R | TTC ATC GGC CAG AAT GAT GAC CAC GAG GTT GAT GAT AAC |
| G558A | F | GTT TAC GCC TGC ATT GCC AGG TGG CTC ACC AAG ATT GAG |
| G558A | R | AAT GCA GGC GTA AAC TTC ATC CAG CAG AAT GAT GAC CAC |
| T581A | F | AGG CTA GCC TTC AAG GCC TTC CTG CTC AAG TTT GTG AAC |
| T581A | R | CTT GAA GGC TAG CCT CTC CTC AAA GCT CTT CTC TGT CTT |
| F585A | F | AAG GCC GCC CTG CTC AAG TTT GTG AAC TCT TAC ACT CCC |
| F585A | R | GAG CAG GGC GGC CTT GAA GGT TAG CCT CTC CTC AAA GCT |

| | | |
|---|---|---|
| F589A | F | CTC AAG GCC GTG AAC TCT TAC ACT CCC ATC TTC TAT GTC |
| F589A | R | GTT CAC GGC CTT GAG CAG GAA GGC CTT GAA GGT TAG CCT |
| V590A | F | AAG TTT GCC AAC TCT TAC ACT CCC ATC TTC TAT GTC GCC |
| V590A | R | AGA GTT GGC AAA CTT GAG CAG GAA GGC CTT GAA GGT TAG |
| N591A | F | TTT GTG GCA TCT TAC ACT CCC ATC TTC TAT GTC GCC TTC |
| N591A | R | GTA AGA TGC CAC AAA CTT GAG CAG GAA GGC CTT GAA GGT |
| S592A | F | GTG AAC GCA TAC ACT CCC ATC TTC TAT GTC GCC TTC TTC |
| S592A | R | AGT GTA TGC GTT CAC AAA CTT GAG CAG GAA GGC CTT GAA |
| Y593A | F | AAC TCT GCC ACT CCC ATC TTC TAT GTC GCC TTC TTC AAA |
| Y593A | R | GGG AGT GGC AGA GTT CAC AAA CTT GAG CAG GAA GGC CTT |
| T594A | F | TCT TAC GCC CCC ATC TTC TAT GTC GCC TTC TTC AAA GGC |
| T594A | R | GAT GGG GGC GTA AGA GTT CAC AAA CTT GAG CAG GAA GGC |
| P595A | F | TAC ACT GCC ATC TTC TAT GTC GCC TTC TTC AAA GGC CGG |
| P595A | R | GAA GAT GGC AGT GTA AGA GTT CAC AAA CTT GAG CAG GAA |
| I596A | F | ACT CCC GCC TTC TAT GTC GCC TTC TTC AAA GGC CGG TTT |
| I596A | R | ATA GAA GGC GGG AGT GTA AGA GTT CAC AAA CTT GAG CAG |
| F597A | F | CCC ATC GCC TAT GTC GCC TTC TTC AAA GGC CGG TTT GTT |
| F597A | R | GAC ATA GGC GAT GGG AGT GTA AGA GTT CAC AAA CTT GAG |
| Y598A | F | ATC TTC GCC GTC GCC TTC TTC AAA GGC CGG TTT GTT GGT |
| Y598A | R | GGC GAC GGC GAA GAT GGG AGT GTA AGA GTT CAC AAA CTT |
| V599A | F | TTC TAT GCC GCC TTC TTC AAA GGC CGG TTT GTT GGT CGG |
| V599A | R | GAA GGC GGC ATA GAA GAT GGG AGT GTA AGA GTT CAC AAA |
| F620A | F | CGC TCT GCC CGG ATG GAG GAG TGT GCC CCG GGC GGC TGC |
| F620A | R | CAT CCG GGC AGA GCG GAA GAT GTA CAC GTA GTC ACC GGG |
| M622A | F | TTC CGG GCC GAG GAG TGT GCC CCG GGC GGC TGC CTC ATG |
| M622A | R | CTC CTC GGC CCG GAA AGA GCG GAA GAT GTA CAC GTA GTC |
| E623A | F | CGG ATG GCC GAG TGT GCC CCG GGC GGC TGC CTC ATG GAG |
| E623A | R | ACA CTC GGC CAT CCG GAA AGA GCG GAA GAT GTA CAC GTA |
| L634A | F | ATG GAG GCC TGT ATC CAG CTG AGC ATC ATT ATG CTG GGC |
| L634A | R | GAT ACA GGC CTC CAT GAG GCA GCC GCC CGG GGC ACA CTC |
| C635A | F | GAG CTC GCC ATC CAG CTG AGC ATC ATT ATG CTG GGC AAG |
| C635A | R | CTG GAT GGC GAG CTC CAT GAG GCA GCC GCC CGG GGC ACA |
| I636A | F | CTC TGT GCC CAG CTG AGC ATC ATT ATG CTG GGC AAG CAG |
| I636A | R | CAG CTG GGC ACA GAG CTC CAT GAG GCA GCC GCC CGG GGC |
| Q637A | F | TGT ATC GCC CTG AGC ATC ATT ATG CTG GGC AAG CAG CTA |
| Q637A | R | GCT CAG GGC GAT ACA GAG CTC CAT GAG GCA GCC GCC CGG |
| L638A | F | ATC CAG GCC AGC ATC ATT ATG CTG GGC AAG CAG CTA ATC |
| L638A | R | GAT GCT GGC CTG GAT ACA GAG CTC CAT GAG GCA GCC GCC |
| S639A | F | CAG CTG GCC ATC ATT ATG CTG GGC AAG CAG CTA ATC CAG |
| S639A | R | AAT GAT GGC CAG CTG GAT ACA GAG CTC CAT GAG GCA GCC |
| I640A | F | CTG AGC GCC ATT ATG CTG GGC AAG CAG CTA ATC CAG AAC |

| | | |
|---|---|---|
| I640A | R | CAT AAT GGC GCT CAG CTG GAT ACA GAG CTC CAT GAG GCA |
| I641A | F | AGC ATC GCC ATG CTG GGC AAG CAG CTA ATC CAG AAC AAT |
| I641A | R | CAG CAT GGC GAT GCT CAG CTG GAT ACA GAG CTC CAT GAG |
| I641C | F | AGC ATC TGT ATG CTG GGC AAG CAG CTA ATC CAG AAC AAT |
| I641C | R | CAG CAT ACA GAT GCT CAG CTG GAT ACA GAG CTC CAT GAG |
| I641F | F | AGC ATC TTT ATG CTG GGC AAG CAG CTA ATC CAG AAC AAT |
| I641F | R | CAG CAT AAA GAT GCT CAG CTG GAT ACA GAG CTC CAT GAG |
| I641M | F | AGC ATC ATG ATG CTG GGC AAG CAG CTA ATC CAG AAC AAT |
| I641M | R | CAG CAT CAT GAT GCT CAG CTG GAT ACA GAG CTC CAT GAG |
| I641N | F | AGC ATC AAC ATG CTG GGC AAG CAG CTA ATC CAG AAC AAT |
| I641N | R | CAG CAT GTT GAT GCT CAG CTG GAT ACA GAG CTC CAT GAG |
| I641V | F | AGC ATC GTG ATG CTG GGC AAG CAG CTA ATC CAG AAC AAT |
| I641V | R | CAG CAT CAC GAT GCT CAG CTG GAT ACA GAG CTC CAT GAG |
| I641Y | F | AGC ATC TAC ATG CTG GGC AAG CAG CTA ATC CAG AAC AAT |
| I641Y | R | CAG CAT GTA GAT GCT CAG CTG GAT ACA GAG CTC CAT GAG |
| M642A | F | ATC ATT GCC CTG GGC AAG CAG CTA ATC CAG AAC AAT CTC |
| M642A | R | GCC CAG GGC AAT GAT GCT CAG CTG GAT ACA GAG CTC CAT |
| L643A | F | ATT ATG GCC GGC AAG CAG CTA ATC CAG AAC AAT CTC TTC |
| L643A | R | CTT GCC GGC CAT AAT GAT GCT CAG CTG GAT ACA GAG CTC |
| G644A | F | ATG CTG GCA AAG CAG CTA ATC CAG AAC AAT CTC TTC GAG |
| G644A | R | CTG CTT TGC CAG CAT AAT GAT GCT CAG CTG GAT ACA GAG |
| Q646A | F | GGC AAG GCA CTA ATC CAG AAC AAT CTC TTC GAG ATT GGC |
| Q646A | R | GAT TAG TGC CTT GCC CAG CAT AAT GAT GCT CAG CTG GAT |
| L647A | F | AAG CAG GCC ATC CAG AAC AAT CTC TTC GAG ATT GGC ATC |
| L647A | R | CTG GAT GGC CTG CTT GCC CAG CAT AAT GAT GCT CAG CTG |
| I648A | F | CAG CTA GCC CAG AAC AAT CTC TTC GAG ATT GGC ATC CCG |
| I648A | R | GTT CTG GGC TAG CTG CTT GCC CAG CAT AAT GAT GCT CAG |
| Q649A | F | CTA ATC GCA AAC AAT CTC TTC GAG ATT GGC ATC CCG AAG |
| Q649A | R | ATT GTT TGC GAT TAG CTG CTT GCC CAG CAT AAT GAT GCT |
| L652A | F | AAC AAT GCC TTC GAG ATT GGC ATC CCG AAG ATG AAA AAG |
| L652A | R | CTC GAA GGC ATT GTT CTG GAT TAG CTG CTT GCC CAG CAT |
| F653A | F | AAT CTC GCC GAG ATT GGC ATC CCG AAG ATG AAA AAG TTC |
| F653A | R | AAT CTC GGC GAG ATT GTT CTG GAT TAG CTG CTT GCC CAG |
| I655A | F | TTC GAG GCC GGC ATC CCG AAG ATG AAA AAG TTC ATC CGC |
| I655A | R | GAT GCC GGC CTC GAA GAG ATT GTT CTG GAT TAG CTG CTT |
| G656A | F | GAG ATT GCA ATC CCG AAG ATG AAA AAG TTC ATC CGC TAC |
| G656A | R | CGG GAT TGC AAT CTC GAA GAG ATT GTT CTG GAT TAG CTG |
| I657A | F | ATT GGC GCC CCG AAG ATG AAA AAG TTC ATC CGC TAC CTG |
| I657A | R | CTT CGG GGC GCC AAT CTC GAA GAG ATT GTT CTG GAT TAG |
| P658A | F | GGC ATC GCA AAG ATG AAA AAG TTC ATC CGC TAC CTG AAG |
| P658A | R | CAT CTT TGC GAT GCC AAT CTC GAA GAG ATT GTT CTG GAT |

| | | |
|---|---|---|
| Y703A | F | CCC GAG GCC ATG GAA ATG ATC ATT CAG TTC GGC TTT GTC |
| Y703A | R | TTC CAT GGC CTC GGG CGT GAG GCC GGC GAA AGG TTC GAG |
| M704A | F | GAG TAC GCC GAA ATG ATC ATT CAG TTC GGC TTT GTC ACC |
| M704A | R | CAT TTC GGC GTA CTC GGG CGT GAG GCC GGC GAA AGG TTC |
| M706A | F | ATG GAA GCC ATC ATT CAG TTC GGC TTT GTC ACC CTG TTT |
| M706A | R | AAT GAT GGC TTC CAT GTA CTC GGG CGT GAG GCC GGC GAA |
| I707A | F | GAA ATG GCC ATT CAG TTC GGC TTT GTC ACC CTG TTT GTT |
| I707A | R | CTG AAT GGC CAT TTC CAT GTA CTC GGG CGT GAG GCC GGC |
| I708A | F | ATG ATC GCC CAG TTC GGC TTT GTC ACC CTG TTT GTT GCG |
| I708A | R | GAA CTG GGC GAT CAT TTC CAT GTA CTC GGG CGT GAG GCC |
| Q709A | F | ATC ATT GCA TTC GGC TTT GTC ACC CTG TTT GTT GCG TCC |
| Q709A | R | GCC GAA TGC AAT GAT CAT TTC CAT GTA CTC GGG CGT GAG |
| F710A | F | ATT CAG GCC GGC TTT GTC ACC CTG TTT GTT GCG TCC TTC |
| F710A | R | AAA GCC GGC CTG AAT GAT CAT TTC CAT GTA CTC GGG CGT |
| G711A | F | CAG TTC GCC TTT GTC ACC CTG TTT GTT GCG TCC TTC CCT |
| G711A | R | GAC AAA GGC GAA CTG AAT GAT CAT TTC CAT GTA CTC GGG |
| F712A | F | TTC GGC GCC GTC ACC CTG TTT GTT GCG TCC TTC CCT CTG |
| F712A | R | GGT GAC GGC GCC GAA CTG AAT GAT CAT TTC CAT GTA CTC |
| V713A | F | GGC TTT GCC ACC CTG TTT GTT GCG TCC TTC CCT CTG GCT |
| V713A | R | CAG GGT GGC AAA GCC GAA CTG AAT GAT CAT TTC CAT GTA |
| T714A | F | TTT GTC GCC CTG TTT GTT GCG TCC TTC CCT CTG GCT CCA |
| T714A | R | AAA CAG GGC GAC AAA GCC GAA CTG AAT GAT CAT TTC CAT |
| L715A | F | GTC ACC GCC TTT GTT GCG TCC TTC CCT CTG GCT CCA CTC |
| L715A | R | AAC AAA GGC GGT GAC AAA GCC GAA CTG AAT GAT CAT TTC |
| F716A | F | ACC CTG GCC GTT GCG TCC TTC CCT CTG GCT CCA CTC TTC |
| F716A | R | CGC AAC GGC CAG GGT GAC AAA GCC GAA CTG AAT GAT CAT |
| F720A | F | GCG TCC GCC CCT CTG GCT CCA CTC TTC GCC CTG CTA AAC |
| F720A | R | CAG AGG GGC GGA CGC AAC AAA CAG GGT GAC AAA GCC GAA |
| F726A | F | CCA CTC GCC GCC CTG CTA AAC AAC ATC ATT GAG ATC CGC |
| F726A | R | CAG GGC GGC GAG TGG AGC CAG AGG GAA GGA CGC AAC AAA |
| L729A | F | GCC CTG GCC AAC AAC ATC ATT GAG ATC CGC CTG GAT GCC |
| L729A | R | GTT GTT GGC CAG GGC GAA GAG TGG AGC CAG AGG GAA GGA |
| N731A | F | CTA AAC GCC ATC ATT GAG ATC CGC CTG GAT GCC AAA AAG |
| N731A | R | AAT GAT GGC GTT TAG CAG GGC GAA GAG TGG AGC CAG AGG |
| I733A | F | AAC ATC GCC GAG ATC CGC CTG GAT GCC AAA AAG TTT GTC |
| I733A | R | GAT CTC GGC GAT GTT GTT TAG CAG GGC GAA GAG TGG AGC |
| I774A | F | GTC ATC GCC AAT GCC TTT GTG ATC TCC TTC ACG TCT GAC |
| I774A | R | GGC ATT GGC GAT GAC AGC CAG CTT CCC AAC ACC TCT GAG |

F, forward; R, reverse

## Supplementary Note

MWC models describe cyclic schemes where closed and open states of an ion channel exist for each level of ligand occupancy[1,2]. A simple mechanistic model of a channel connecting three closed and open states with distinct ligand occupancy is shown below:

$$\begin{array}{ccccc} & & L_0 & & \\ & C_0 & \leftrightarrow & O_0 & \\ K_{d(C)} & \updownarrow & & \updownarrow & K_{d(O)} \\ & C_1 & \leftrightarrow & O_1 & \\ K_{d(C)} & \updownarrow & & \updownarrow & K_{d(O)} \\ & C_2 & \leftrightarrow & O_2 & \\ & & L_2 & & \end{array}$$

Each state is characterized by an energy level and the equilibrium occupancy of these states is governed by the Boltzmann distribution,

$$p_i = \frac{e^{-G_i/RT}}{\sum_j e^{-G_j/RT}}$$

where $p_i$ denotes the occupancy of state $i$, $G_i$ the corresponding free energy, and $i \in j$. States with lower energy are occupied with higher probability.

For the above mechanism,

$$P_O = \frac{L_0 Q_O}{Q_C + L_0 Q_O} \tag{A1}$$

where

$$Q_C = 1 + \frac{x}{K_{d(C)}} + \left(\frac{x}{K_{d(C)}}\right)^2$$

$$Q_O = 1 + \frac{x}{K_{d(O)}} + \left(\frac{x}{K_{d(O)}}\right)^2$$

where $P_O$ is the open probability, $K_d$ defines the equilibrium dissociation constant, $L$ the efficacy of the transition, and $x$ the ligand concentration. It is thus clear that the protein's response to its ligand is governed by both the binding affinity and the isomerization constants. The mean current response ($\bar{\imath}$) is given by

$$\bar{\imath} = \sum_j i_j p_j$$

where $i_j$ is the unitary current of state $j$ and $p$ is defined as above. Because the energy difference between the initial state and the final state is constant regardless of the path that is taken in the cycle, the following well-known condition holds

$$K_{d(C)}{}^2/K_{d(O)}{}^2 = L_2/L_0$$

For activation to occur,

$$\frac{L_2}{L_0} > 1$$

$$\frac{K_{d(C)}}{K_{d(O)}} > 1$$

which states that the binding affinity of the open state is necessarily higher than that of the closed state. In general, the apparent affinity of activation, typically quantified using the $EC_{50}$, adopts a value that is bounded by the intrinsic binding affinity of the closed and the open states. A shift in the efficacy would result in a shift in the apparent affinity towards the intrinsic affinity of the closed or the open states depending on the direction of the shift.

We illustrate these properties through simple calculations using the above mechanism (Appendix Fig. 1). In this study, we observed that the non-conductive conformation of the pore in the closed state is stabilized by hydrophobic interactions between bulky aliphatic residues at the inner end of the pore. We incorporated this effect by assuming that the gating constant $L_2$ can be expressed as a function of the number of methyl groups ($n_{\mathrm{Me}}$) and that on average each methyl group has an identical effective energetic contribution ($\Delta G_{\mathrm{Me}}$)

$$L_2(\Delta n_{\mathrm{Me}}) = L_{2_{\mathrm{WT}}} e^{-\Delta n_{\mathrm{Me}} \Delta G_{\mathrm{Me}}/RT} \quad \text{(A2)}$$

where

$$\Delta n_{\mathrm{Me}} = n_{\mathrm{Me(mut)}} - n_{\mathrm{Me(WT)}}$$

From the calculations, it can be observed that increasing efficacy by methyl truncation shifts the $EC_{50}$ towards the left and concomitantly increases occupancy of the open state in the absence of $Ca^{2+}$, while the converse applies for a decrease in efficacy (Appendix Fig. 1a). Within the investigated range, the $EC_{50}$ shifts from the intrinsic affinity of the closed state to that of the open state as efficacy increases but saturates on both ends of the spectrum (Appendix Fig. 1b), as is observed in our experiments (Fig. 4a, c). When the efficacy becomes excessively high ($\Delta n_{\mathrm{Me}} = -6$), the system is reduced to one that consists of effectively only the open states and the current response corresponds to the transition from the low-conductance open state $O_0$ to the high-conductance open state $O_2$ (Appendix Fig. 1c). The apparent affinity thus corresponds closely to that of the open states as the contribution of the closed states is minimal. This is also evident in Eq. A1, where the gating constant $L_2$ dictates the relative contribution of the binding polynomials $Q_C$ and $Q_O$. In the case where the efficacy is more balanced ($\Delta n_{\mathrm{Me}} = 0$), the system consists of

first mainly the closed states at lower $Ca^{2+}$ concentrations but which becomes dominated by the open states as the $Ca^{2+}$ concentration increases (Appendix Fig. 1d). As a result, the $EC_{50}$ lies between the high and the low affinity limits and an affinity shift is resulted which underlies the apparent positive cooperativity even when the successive binding steps are assumed to not functionally interact. The same principles apply for cases where the efficacy becomes very low ($\Delta n_{\mathrm{Me}} = 6$) (Appendix Fig. 1e).

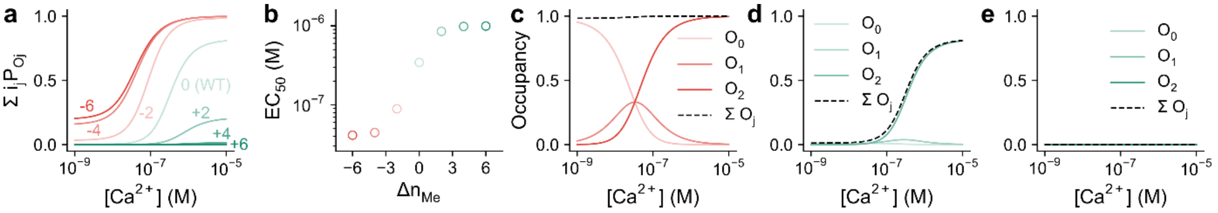


**Appendix Fig. 1: Properties of the model. a,b** Concentration-response relation (**a**) and $EC_{50}$ (**b**) as a function of efficacy. **c-e** Occupancy of the open states $O_0$, $O_1$, $O_2$, and their sum as a function of $Ca^{2+}$ concentration when efficacy is excessively high ($\Delta n_{\mathrm{Me}} = -6$) (**c**), balanced ($\Delta n_{\mathrm{Me}} = 0$) (**d**), and very low ($\Delta n_{\mathrm{Me}} = 6$) (**e**). $\Delta n_{\mathrm{Me}}$ is the change in the number of methyl groups, which is related to efficacy via Eq. A2 in the presented model.

In addition to the general trend observed for methyl truncation, we observed effects specific to the individual mutants and non-additivity amongst them. We therefore allowed, instead of the strict dependence on the number of methyl groups, each mutation to exert a specific effect

$$L_{2_{\mathrm{mut}}} = L_{2_{\mathrm{WT}}} e^{-\Delta G_{\mathrm{mut}}/RT} \quad \text{(A3)}$$

We illustrate the robustness of this analysis by quantifying the errors of the estimated parameters, given the precision of the experimental data, using a simple Monte Carlo approach (Appendix Fig. 2). Random shifts drawn from the normal distribution using the standard deviation specific to each data point were applied to the mean data to mimic random sampling during an experiment. These synthetic datasets were then fitted with shared parameters $K_{d(C)}$ and $K_{d(O)}$, while the mutant-specific parameters $\Delta G_{\mathrm{mut}}$ were allowed to vary independently. This sampling procedure was then repeated 1000 times to obtain a distribution for each parameter. From these simulations, we observed that the 95% confidence intervals (CI) are finite and the estimates are centred at the most probable value of the parameter (Appendix Fig. 2a). Although correlation between some parameters is inevitable in a multi-parameter space (Appendix Fig. 2b), the distributions are sufficiently narrow (95% CI <11% of the estimates and ~18% for $K_{d(O)}$) for a meaningful interpretation. As expected, similar values were obtained for the shared parameter set ($K_{d(C)}, K_{d(O)}$) regardless of whether the gating constant $L_2$ is expressed as a function of a shared parameter ($\Delta G_{\mathrm{Me}}$ in Eq. A2) or of a mutant-specific parameter ($\Delta G_{\mathrm{mut}}$ in Eq. A3). The incorporation of mutant-specific effects in the latter, however, allows the deviation of the $EC_{50}$'s

from the general trend (Fig. 4c) to be accounted for, therefore addressing the inadequacy of using a single parameter $\Delta G_{\mathrm{Me}}$ for the investigated mutants.

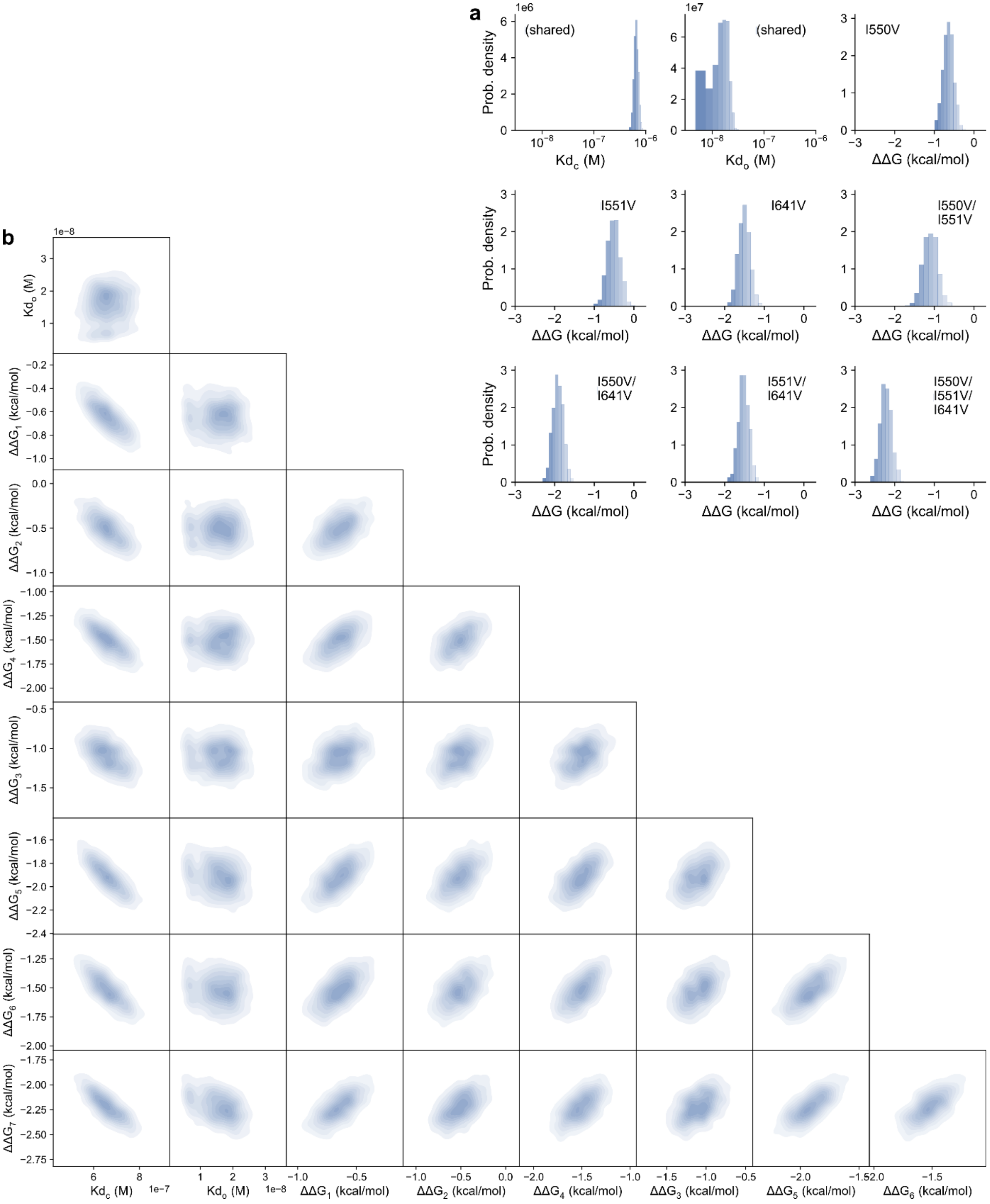

**Appendix Fig. 2: Monte Carlo confidence intervals and parameter distribution. a** Histograms of parameters estimated from 1000 independent fits with random shifts applied to the mean data. The shifts were drawn from the normal distribution using the standard deviation specific to each data point. **b** Pairwise correlation between the best-fit values. $\Delta\Delta G_{1-7}$ correspond to the estimated values for I550V, I551V, I641V, I550V/I551V, I550V/I641V, I551V/I641V, and I550V/I551V/I641V respectively.

## References


1 Monod, J., Wyman, J. & Changeux, J. P. On the Nature of Allosteric Transitions: A Plausible Model. *J Mol Biol* **12**, 88-118, doi:10.1016/s0022-2836(65)80285-6 (1965).

2 Auerbach, A. Thinking in cycles: MWC is a good model for acetylcholine receptor-channels. *J Physiol* **590**, 93-98, doi:10.1113/jphysiol.2011.214684 (2012).
