# Supplementary material for: Gating the pore of the calcium-activated chloride channel TMEM16A
Source: Nat Commun. 2021 Feb 4;12:785. doi: 10.1038/s41467-020-20787-9 (PMC7862301; doi:10.1038/s41467-020-20787-9)
Supplement: Supplementary file 3 — Reporting Summary [file 41467_2020_20787_MOESM3_ESM.pdf]

nature research

Corresponding author(s): Raimund Dutzler
Andy K.M. Lam
Cristina Paulino

Last updated by author(s): Dec 1, 2020

# Reporting Summary

Nature Research wishes to improve the reproducibility of the work that we publish. This form provides structure for consistency and transparency in reporting. For further information on Nature Research policies, see our Editorial Policies and the Editorial Policy Checklist.

## Statistics

For all statistical analyses, confirm that the following items are present in the figure legend, table legend, main text, or Methods section.

| n/a | Confirmed | |
|---|---|---|
| ☐ | ☒ | The exact sample size ($n$) for each experimental group/condition, given as a discrete number and unit of measurement |
| ☐ | ☒ | A statement on whether measurements were taken from distinct samples or whether the same sample was measured repeatedly |
| ☐ | ☒ | The statistical test(s) used AND whether they are one- or two-sided<br>*Only common tests should be described solely by name; describe more complex techniques in the Methods section.* |
| ☐ | ☒ | A description of all covariates tested |
| ☐ | ☒ | A description of any assumptions or corrections, such as tests of normality and adjustment for multiple comparisons |
| ☐ | ☒ | A full description of the statistical parameters including central tendency (e.g. means) or other basic estimates (e.g. regression coefficient) AND variation (e.g. standard deviation) or associated estimates of uncertainty (e.g. confidence intervals) |
| ☐ | ☒ | For null hypothesis testing, the test statistic (e.g. $F$, $t$, $r$) with confidence intervals, effect sizes, degrees of freedom and $P$ value noted<br>*Give P values as exact values whenever suitable.* |
| ☒ | ☐ | For Bayesian analysis, information on the choice of priors and Markov chain Monte Carlo settings |
| ☒ | ☐ | For hierarchical and complex designs, identification of the appropriate level for tests and full reporting of outcomes |
| ☒ | ☐ | Estimates of effect sizes (e.g. Cohen's $d$, Pearson's $r$), indicating how they were calculated |

*Our web collection on statistics for biologists contains articles on many of the points above.*

## Software and code

Policy information about availability of computer code

| | |
|---|---|
| Data collection | Clampex 10.6, EPU v2.4, Digital Micrograph 3.23 |
| Data analysis | Clampfit 10.7, Excel 2019, Prism 8, NumPy 1.18.5, SciPy 1.5.0, Focus v1.1.0, MotionCorr2 v1.2.1, CTFFIND 4.1.13, Relion 3.0.8, cryoSPARC v2.15, Phenix 1.16, Coot 0.8.9.2, HOLE 2.2.004, crYOLO 1.4.1, UCSF Chimera 1.13.1, ChimeraX 0.93, VMD 1.9.4 |

For manuscripts utilizing custom algorithms or software that are central to the research but not yet described in published literature, software must be made available to editors and reviewers. We strongly encourage code deposition in a community repository (e.g. GitHub). See the Nature Research guidelines for submitting code & software for further information.

## Data

Policy information about availability of data

All manuscripts must include a data availability statement. This statement should provide the following information, where applicable:

- Accession codes, unique identifiers, or web links for publicly available datasets
- A list of figures that have associated raw data
- A description of any restrictions on data availability

Maps, half-maps and masks have been deposited in the EMDB and can be found under the entries EMD-12025 (WT-Ca2+), EMD-12026 (I551A-apo), EMD-12027 (I551A-Ca2+). The respective atomic models are available in the PDB under 7B5C (WT-Ca2+), 7B5D (I551A-apo), 7B5E (I551A-Ca2+). Electrophysiology data that support the findings of this study are available from the corresponding authors upon reasonable request.

# Field-specific reporting

Please select the one below that is the best fit for your research. If you are not sure, read the appropriate sections before making your selection.

☒ Life sciences ☐ Behavioural & social sciences ☐ Ecological, evolutionary & environmental sciences

For a reference copy of the document with all sections, see nature.com/documents/nr-reporting-summary-flat.pdf

# Life sciences study design

All studies must disclose on these points even when the disclosure is negative.

| | |
|---|---|
| Sample size | No sample size determination was performed. Experiments were performed multiple times with similar results and further inclusion of data does not change the results. |
| Data exclusions | Leaky recordings were discarded, and otherwise no data were excluded from the analyses. |
| Replication | Elecctrophysiology data show the mean or median of the indicated number of biological replicates and errors are indicated. Experiemnts were performed multiple times from different transfections. All replications were successful. |
| Randomization | Not applicable as it is not relevant in the study. |
| Blinding | Not applicable as this is deemed not practically feasible. |

# Reporting for specific materials, systems and methods

We require information from authors about some types of materials, experimental systems and methods used in many studies. Here, indicate whether each material, system or method listed is relevant to your study. If you are not sure if a list item applies to your research, read the appropriate section before selecting a response.

## Materials & experimental systems

| n/a | Involved in the study | |
|---|---|---|
| ☒ | ☐ | Antibodies |
| ☐ | ☒ | Eukaryotic cell lines |
| ☒ | ☐ | Palaeontology and archaeology |
| ☒ | ☐ | Animals and other organisms |
| ☒ | ☐ | Human research participants |
| ☒ | ☐ | Clinical data |
| ☒ | ☐ | Dual use research of concern |

## Methods

| n/a | Involved in the study | |
|---|---|---|
| ☒ | ☐ | ChIP-seq |
| ☒ | ☐ | Flow cytometry |
| ☒ | ☐ | MRI-based neuroimaging |

## Eukaryotic cell lines

Policy information about cell lines

| | |
|---|---|
| Cell line source(s) | HEK293T (ATCC CRL-1573) , HEK293S GnTI- (ATCC CRL-3022) |
| Authentication | As these are commercially available cell lines, no further cell line authentication was performed. |
| Mycoplasma contamination | The cell lines were tested and are free from mycoplasma contamination. |
| Commonly misidentified lines (See ICLAC register) | No commonly misidentified cell lines were used in the study |
